# Hyperspectral Monitoring of Green Roof Vegetation Health State in Sub-Mediterranean Climate: Preliminary Results

**DOI:** 10.3390/s17040662

**Published:** 2017-03-23

**Authors:** Patrizia Piro, Michele Porti, Simone Veltri, Emanuela Lupo, Monica Moroni

**Affiliations:** 1 DINCI (Dipartimento di Ingegneria Civile), University of Calabria, via P. Bucci 42B, 87036 Rende (CS), Italy; patrizia.piro@unical.it (P.P.); simone.veltri@unical.it (S.V.); 2 DIMEG (Dipartimento di Ingegneria Meccanica, Energetica e Gestionale), University of Calabria, via P. Bucci 42B, 87036 Rende (CS), Italy; michele.porti@unical.it (M.P.); emanuela.lupo@uniroma1.it (E.L.); 3 DICEA (Dipartimento di Ingegneria Civile Edile e Ambientale), Sapienza University of Rome, via Eudossiana 18, 00184 Rome, Italy

**Keywords:** hyperspectral monitoring, vegetation indices, green roofs

## Abstract

In urban and industrial environments, the constant increase of impermeable surfaces has produced drastic changes in the natural hydrological cycle. Decreasing green areas not only produce negative effects from a hydrological-hydraulic perspective, but also from an energy point of view, modifying the urban microclimate and generating, as shown in the literature, heat islands in our cities. In this context, green infrastructures may represent an environmental compensation action that can be used to re-equilibrate the hydrological and energy balance and reduce the impact of pollutant load on receiving water bodies. To ensure that a green infrastructure will work properly, vegetated areas have to be continuously monitored to verify their health state. This paper presents a ground spectroscopy monitoring survey of a green roof installed at the University of Calabria fulfilled via the acquisition and analysis of hyperspectral data. This study is part of a larger research project financed by European Structural funds aimed at understanding the influence of green roofs on rainwater management and energy consumption for air conditioning in the Mediterranean area. Reflectance values were acquired with a field-portable spectroradiometer that operates in the range of wavelengths 350–2500 nm. The survey was carried out during the time period November 2014–June 2015 and data were acquired weekly. Climatic, thermo-physical, hydrological and hydraulic quantities were acquired as well and related to spectral data. Broadband and narrowband spectral indices, related to chlorophyll content and to chlorophyll–carotenoid ratio, were computed. The two narrowband indices NDVI_705_ and SIPI turned out to be the most representative indices to detect the plant health status.

## 1. Introduction

Green infrastructure refers to a suite of technologies, such as green roofs, permeable pavements, rain gardens, and bioswales. Through the combination of technologies and vegetated areas, they absorb and slow down the flow of stormwater from impervious urban surfaces (streets, parking lots, rooftops, and walkways) that dominate an urban watershed [1,2,3]. Well-designed and strategically sited green infrastructure can reduce the rate and volume of urban runoff, reducing the need for costly new or expanded wastewater treatment facilities. Other remarkable advantages of green infrastructures are: their ornamental role, reduction of noises and improvement of thermal effects within the building in which they are installed. Furthermore, green infrastructures contribute to the diminishment of the urban heat island effects, reducing temperatures via evapotraspiration [4] and guaranteeing biodiversity in the urban environment, representing a novel habitat available for plants and animals.

High temporal resolution monitoring systems are mandatory in order to check the health state of vegetated areas and ensure that a green infrastructure will work properly.

A variety of sensing methodologies have been set up to detect the vegetation health state. Sensing techniques allow the detection of physiological abnormalities of the vegetation even prior to the appearance of visual symptoms. For this reason, they are particularly suitable to highlight stressful situations (i.e., water stress, air pollutants and presence of soil contaminants) that impact the plant chemical and physiological processes. Among sensing techniques, spectroscopy prescribes the acquisition of vegetation spectral reflectance, which is a source of information on its chemical-physical status and physiological properties. It is observed that the reflectance of stressed plants has different characteristics compared to the typical reflectance of healthy vegetation. The limitations of spectroscopy applications are the cost of the equipment and the management of the amount of data in high resolution applications [5]. Nevertheless, spectroscopy is the most flexible, efficient and established technology to fully characterize the vegetation health state compared to the other sensing techniques, mainly when combined with aerial platforms (such as helicopters, balloons and cranes).

Spectral measurements can be obtained by multispectral or hyperspectral devices. The spectral resolution is related to the number of bands that can be acquired and to their bandwidth. The sensors are multispectral, if they record a limited number of different bands (up to 10), large and usually not contiguous, and hyperspectral, if they can detect hundreds of spectral bands that are generally very narrow and closely spaced.

Several platforms can be employed for field hyperspectral investigations, either via aerial devices (satellites, helicopters, balloons, unmanned aerial platforms (UAP)) or fixed structures (tripods). Remarkable geometric and spectral resolutions have been achieved in recent years by earth satellite observation systems, such as Hyperion and WorldView-3. The Hyperion System is a sensor with a high spectral resolution. It records 220 bands between 400 nm and 2500 nm, with a spatial resolution of 30 m [6]. Images of the multispectral system WorldView-3 presents high spatial resolution, i.e., 1.24 m. The Environmental Mapping and Analysis Program (EnMAP) is a German hyperspectral satellite mission with a spatial resolution of 30 m × 30 m. Examples of airborne hyperspectral sensors include Airborne Visible/Infrared Imaging Spectrometer (AVIRIS), Compact Airborne Spectrographic Imager (CASI), HySpex NEO Hyperspectral cameras and SPECIM AISA with spatial resolution of 17 m, from sub-meters to 10 meters, depending on the close-up lenses, of 1.1 m or 1.5 m at 1000 m altitude, respectively [6]. Higher spatial resolutions (up to centimetres) may be achieved using linear spectrometers mounted on ultralight aircraft [7] or multispectral sensors on board a UAP [8].

For even higher resolutions, ground spectroscopy with the equipment, either tunable filters or spectroradiometers, mounted on a fixed stand is preferred. When interference tunable filters are employed, it is necessary to acquire more pictures and tune each filter wavelength to gather the full spectrum of the scene [9]. Spectroradiometer delivers the fastest and most accurate spectral field measurements, acquiring a broad spectral range in a tenth of a second [10]. With respect to proximal or remote sensing, several errors can be minimized with ground spectroscopy, since the measurements are taken a short distance away from the surface under investigation, and data calibration is more accurate when calibrated Lambertian panels are employed.

The study of the spectral signature of vegetation, using ground, proximal and remote sensing techniques, is a well-established method of environmental monitoring. Urban remote sensing applied to map urban land cover at various levels of detail, in particular vegetated areas in urban environments, is presented in [11]. Vegetation reflectance was studied to detect the response to numerous stress agents, including ozone, pathogens, senescence, dehydration [12], natural gas [13] and metal contamination [14]. The increased reflectance in the visible region (VIS) and the reduced reflectance in the near-infrared region (NIR) were found to be consistent with chlorophyll reduction and cell structure damage among various species due to stress agents [12,14,15]. These changes in VIS and NIR were also found in vegetation responses to stress due to salt [16,17]. Ref. [18] applied the normalised difference vegetation index to discriminate different vegetation in urban and suburban areas. Ref. [9], among others, assessed the health state of vegetation in an industrial area affected by contamination via suitable indices (Vegetation Indices, i.e., VIs) evaluated through a combination of bands sensitive or not to stress.

This paper presents a ground spectroscopy sensing monitoring survey of a green roof installed at the University of Calabria fulfilled via the acquisition and analysis of hyperspectral data. This study is part of a larger research project “Integrated and Sustainable Management Services for the Water-Energy Cycle in Urban Drainage Systems” financed by European Structural funds aimed at understanding the influence of green roofs on rainwater management and energy consumption for air conditioning in the Mediterranean area. To this purpose, the study area was equipped with suitable devices for monitoring of climatic, thermo-physical, hydrological and hydraulic quantities. Reflectance values were acquired with a field-portable spectroradiometer (FieldSpec 4–ASD, Analytical Spectral Devices Inc., Boulder, CO, USA), which operates in the range of wavelength 350–2500 nm. The condition of plants was visually inspected and annotated to be compared to the analysis outcomes in addition to meteo-climatic quantity data.

The aim of the survey is (i) to demonstrate that ground spectroscopy may be employed to assess the health state of plants used in the Mediterranean environment for green roofs and (ii) to determine the most appropriate vegetation indices to be employed for an eventual real-time close-range sensing via high spatial resolution multispectral or hyperspectral ground- or air-platforms.

The Triangular Vegetation Index (TVI) was selected as the broadband index and compared with the simple NarrowBand Ratio index (NBR) and the narrowband Normalized Difference Vegetation Index (NDVI_705_). These three indices are related to the chlorophyll content. Two narrowband indices related to the chlorophyll–carotenoid ratio, i.e., the Structure-Insensitive Pigment Index (SIPI) and the simple ratio index Pigment Specific Simple Ratio (PSSR), were further tested as plant health status indicators.

The paper is organized as follows: Section 2 describes the study area and data acquisition, and reviews the vegetation hyperspectral features and the most commonly employed vegetation indices; Section 3 describes the results of the analysis. The paper ends with a concluding section.

## 2. Materials and Methods

### 2.1. Study Area

**Figure 1:**
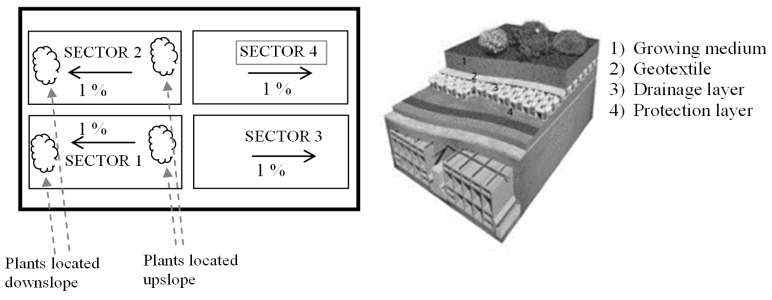
Green roof organization and stratigraphy.

To fulfill the aims of the research project “Integrated and Sustainable Management Services for the Water-Energy Cycle in Urban Drainage Systems”, a scale model of a green roof was installed at the University of Calabria (Figure 1). A typical green roof stratigraphy consists of: a waterproof and anti-root layer, a mechanical protection for the anti-root layer, an integrated layer for drainage/aeration/accumulation, and a growing medium (soil) layer. This medium layer is typically a low-weight combination of sand, aggregate, and organic matter (Figure 1).

In order to facilitate the comparison of different design solutions, the test site was organized in four sectors. Sector 1 (54 m^2^) and sector 2 (51 m^2^) were designed with the same vegetation species but different thickness of the growing medium, the thickness of sector 1 being lower than sector 2. Sector 3 (42 m^2^) and sector 1 had the same stratigraphy, but sector 3 was not vegetated. Sector 4 (39 m^2^) was realized as a conventional roof, equipped with the classic waterproof membrane, and, for this reason, it is considered as a reference. The four sectors have a slope of 1% and are hydraulically independent.

The green roof is equipped with an irrigation network with a drip system to supply water to the plants in case of need.

**Figure 2:**
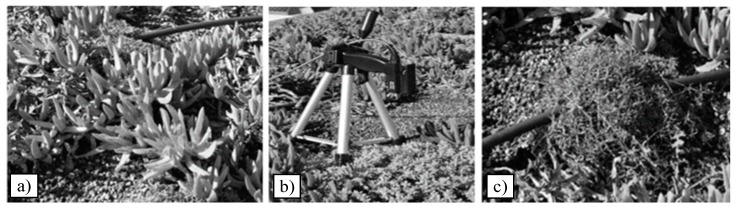
Plant species monitored: (**a**) *Carpobrotus edulis*; (**b**) *Cerastium tomentosum* and (**c**) *Dianthus granthianopolitanus* located upslope in sector 1.

Figure 2 shows the vegetation species investigated, i.e., *Carpobrotus edulis* (hereinafter Carpobrotus), *Cerastium tomentosum* (hereinafter Cerastium) and *Dianthus granthianopolitanus* (hereinafter Dianthus). These species have been chosen during the design of the roof so that they were resistant to local weather conditions, with strong periods of drought, and with a horizontal root development for the limited thickness of the soil (8 cm).

### 2.2. Data Acquisition

The paper presents data measured weekly from November 2014 to June 2015, a sufficiently long period during which plants were affected by outdoor weather conditions and changed their health state. Therefore, the complete annual spectral behavior of the selected species is not available. Nevertheless, the purposes of the investigation have been fully reached. Stress is mainly due to weather conditions and water scarcity. Other causes of stress can be reasonably neglected: there is no deficiency of nutrients as demonstrated by a specific ongoing analysis of organic matter and nitrogen compound soil concentrations [19]. No other diseases have been detected.

Data collection involved sector 1 and sector 2. For each green roof sector, one canopy of each plant species located upslope and one canopy of each plant species located downslope, for a total of six plants, were monitored. The aim was to correlate the plant health state with growing medium thickness, slope and hydraulic configuration of the sector.

For each measurement, the spectroradiometer FieldSpec 4–ASD collected 2151 data points covering the entire spectral range (350–2500 nm) with a resolution of 1 nm. It was mounted at a height of 0.15 m above the foliage to ensure a large field of view including the plant only (0.005 m^2^) (Figure 2). Calibration and optimization were conducted before each spectral measurement using a Spectralon panel as the white reference. For each sample, three spectra were collected and then averaged in order to detect one representative spectral measurement for plant and reduce noise on the reflectance data. Spectral data were measured between 11:30 a.m. and 1:00 p.m. on clear days.

The plants’ condition was visually inspected and annotated to be correlated to the computed spectral indices. Since determination of the plant health status by visual assessment is subjective and varies according to the genus, visual checks were made by personnel with horticulture skills able to associate phenotyping (i.e., characteristic physical traits of a plant) with the plant status. Therefore, the “ground truth” definition was the aim of the field survey presented herein. In fact, the use of the field-portable spectroradiometer allows direct reflectance measurements that can be used as ground truth to validate and calibrate hyperspectral data acquired with ground- or air-platforms [20].

Thermal and weather parameters such as soil temperature at different depth, solar radiation, air temperature, air relative humidity, wind speed and rainfall were recorded during the same sampling time by the monitoring system with which the green roof is equipped. These parameters provide useful information about climate and environmental conditions that can influence the eco-physiological state of the plant, causing changes in its conditions that can be revealed by the hyperspectral system. In order to simplify the result presentation, in this contribution, spectral data will be related to air temperature (in °C), rainfall (in mm/day) and solar radiation (in MJ/(m^2^day)). These data were collected every minute. The temperature is then averaged over 24 h, rainfall is the integral along the day and solar radiation is the integral of the solar irradiance over the daily equivalent hour of full sunlight. Air temperature data from 29 January 2015 to 20 March 2015 are lacking due to the sensor failure.

### 2.3. Vegetation Hyperspectral Features

The canopy spectral signature from the diffusely reflected radiation is described by the ratio of the intensity of reflected light to that of the incoming light for each wavelength in the visible (400–750 nm), near-infrared (750–1200 nm) and shortwave infrared (1200–2500 nm) spectral regions. The typical spectral signature of vegetation is characterized by two clear peaks at 540 nm (Green Peak) and in the near infrared region 700/720 nm (Red Edge) by two minima in correspondence with the absorption peaks of the pigments (chlorophyll well) and the so-called NIR plateau in the near infrared wavelengths 720–1200 nm. The healthy vegetation shows absorption peaks around the wavelengths 420 nm, 490 nm and 660 nm. The red edge is a feature of the plant spectral signature: it is positioned between 690 nm and 720 nm, where the reflectance changes from low values, due to chlorophyll absorption at the red wavelengths of the visible region (chlorophyll well), to higher values in the near infrared region (NIR plateau), associated to the internal structure of the leaf and to the water content. In the near infrared region, the canopies are generally characterized by higher values of reflectance and transmittance than in the visible region. With increasing wavelengths of up to 2500 nm, the reflectance decreases gradually because of increased absorption by the water present in the leaves [21].

Stressful situations, as well as plant senescence, modify the spectral response of vegetation. In the phase of plant senescence, chlorophyll degrades faster than carotene. This entails a significant increase in the visible red area reflectance (600–700 nm), with a measurable decrease of chlorophyll absorption (at 420 nm, 490 nm and 660 nm). In this situation, carotenoids and xanthophylls become dominant pigments in the leaves, which appear yellow (chlorosis) because these chemical compounds absorb blue light while reflecting the green and red parts of the visible spectrum. A combination of green and red provides the yellow color observed. When the leaf dies, brown pigments (tannins) appear, reducing the reflectance in the spectral range 400–750 nm [22].

The red edge position changes in response to an increase or a decrease in the amount of chlorophyll. In particular, a shift towards lower wavelengths means a decrease of chlorophyll content, which can be associated with an unhealthy condition for the plant.

The leaf maturation sees a gradual increase in the reflection in the NIR, while, during the senescence reflectance in the NIR, first increases and then decreases when cells deteriorate [22].

### 2.4. Spectral Indices Selection

Spectral signature of leaves or canopies may be used to quantify vegetation indices. These are the result of mathematical operations between reflectance values both in the visible and near-infrared portions of the spectrum. VIs are used to capture essential characteristics of the spectra, in order to reduce a large volume of data and as indicators of plant pigment content linked to their eco-physiological state.

Canopy reflectance in the visible and near infrared regions is strongly dependent on biochemistry, such as chlorophyll and carotenoid content, and structural properties of the canopy as leaf area index, leaf orientation, canopy structure. Hence, it is impossible to develop a “unique” VI suitable to reveal the health status of each kind of plant. For this reason, a large selection of VI is investigated and the ones more suitable to reveal the health state of the vegetation species investigated (Figure 2) are considered in more detail.

Three primary types of indices have been developed for plant stress estimation: Simple Ratio (SR), Normalized Difference (ND) and Combinations of Reflectance (CoR) values at key wavelengths. An SR index is defined as the ratio between the reflectance values at a reference wavelength (R_ref_) and at an index wavelength (R_index_). Indices based on ND use the same wavelengths as the SR but subtract, rather than divide, R_index_ from R_ref_. Then, the value is normalized through division by the sum of the reflectance at the same two wavelengths. The CoR indices combine in linear expressions or ratio reflectance values at reference and index wavelengths.

Key wavelengths are chosen according to chlorophyll or carotenoid vs. chlorophyll contents, or chlorophyll–carotenoid ratio. For the definition of the former, it must be recalled that the chlorophylls have strong absorbance peaks in the red and blue regions of the spectrum. Since the blue peak (470 nm) overlaps with the absorbance of the carotenoids, it is not generally used for the estimation of chlorophyll content. The maximum absorbance in the red region occurs between 660 and 680 nm. However, reflectance at these wavelengths has proved to be useless for the prediction of chlorophyll content because relatively low chlorophyll contents are sufficient to saturate absorption in the 660–680 nm region, thus reducing sensitivity to high chlorophyll contents of spectral indices based on these wavelengths. Consequently, empirical models for the prediction of chlorophyll content from reflectance are largely based on reflectance in the 550 or 700 nm regions where higher chlorophyll contents are required to saturate absorbance [23,24,25,26,27].

Indices based on carotenoids vs. chlorophyll content were introduced considering that the estimation of carotenoid content from reflectance is more difficult than the estimation of chlorophyll because of the overlap between the chlorophyll and carotenoid absorption peaks, as already specified above, and because of the higher concentration of chlorophyll with respect to carotenoids in most leaves. References [24,28] present attempts to estimate total carotenoids from reflectance; however, these indices have not shown good generality when applied to other data sets [29]. Most indices based on the estimation of the chlorophyll–carotenoid ratio compare reflectance in the region of the carotenoid absorption peak (400–500 nm) with reflectance in the red region, which is influenced by chlorophyll only [30,31].

VIs may also be classified as broadband and narrowband indices. Broadband vegetation indices take into account wavelengths from Green, Red, VIS and NIR bands, as vegetation shows specific reflectance properties in these bands. These properties can reveal the health state of the canopy. Nevertheless, ecosystem features may not be identified with the appropriate level of detail. This led to the development of narrowband indices, requiring a more accurate calibration of the measuring instruments and providing more detailed information. The narrowband indices are based on specific wavelengths associated with the leaf pigments since a change in wavelength values can be related to the physiological state of the plant.

Table 1 presents the definition of the broadband and narrowband vegetation indices based on chlorophyll content and carotenoid vs. chlorophyll content employed in this contribution. In the table, R refers to reflectance and the subscripts refer to specific spectral bands or wavelengths (i.e., NIR refers to the average in the band interval 750–1100 nm, RED to the average in the band interval 600–700 nm and GREEN to the average in the band interval 500–600 nm; w refers to a narrow wavelength w, for instance R_750_ is reflectance at wavelength 750 nm).

In literature, several indices are used to describe the vegetation health, but there are no studies on green roofs in a Mediterranean environment. A larger number of indices were considered, namely, 15 indices, of which five broadband (i.e., Ratio Vegetation Index, Difference Vegetation Index, TVI, NDVI, Renormalized Difference Vegetation Index) and three narrowband (i.e., two-wavelength combinations of NBR, NDVI_705_) indices based on chlorophyll content, and seven narrowband indices based on chlorophyll–carotenoid ratio (i.e., SIPI, three-wavelength combinations of the Pigment Specific normalized difference index, three-wavelength combinations of PSSR). A basic statistical analysis was conducted to evaluate which indices were more sensitive to the health state of the plants on the green roof under investigation, i.e., the ones presenting the larger variability with time.

TVI is a broadband index related to the chlorophyll content. TVI describes the proportion of energy absorbed by the pigments as a function of the differences between reflectance in the green, red and NIR regions. It decreases in the case of vegetation subject to a state of stress [32].

NBR is a narrowband index related to the chlorophyll content. NBR decreases in the case of vegetation subject to a state of stress [9].

NDVI_705_ is a narrowband normalized difference index related to the chlorophyll content. NDVI_705_ is a modified version of the broadband index counterpart. It considers a narrow band at the edge of the chlorophyll absorption peak (at 705 nm) and normalizes the ratio with a wavelength that is not influenced by the chlorophyll content (i.e., 750 nm) [33]. NDVI_705_ decreases when the vegetation is subject to a state of stress.

PSSR and SIPI are two narrowband indices related to the chlorophyll–carotenoid ratio. They are based on the increase observed in the relative concentration of carotenoids compared to chlorophyll when the plants are under stress and in senescence. In particular, in various plant species, a high correlation between both the ratios of the two pigments and the reflectance in the domain of blue (where both carotenoids and chlorophyll absorb) and red (where only chlorophyll absorbs) exists [27].

PSSR is defined as the ratio between a carotenoid content sensitive band (470 nm) and an insensitive band (800 nm). Spectral measurements conducted on healthy vegetation provide lower values of the index compared to vegetation in a state of stress.

SIPI minimizes the effects of radiation interactions at the leaf surface and the internal structure via the introduction of the near infrared spectral band R_800_. Wavelengths 680 nm and 445 nm, empirically selected, correspond to the absorption maxima of chlorophyll-a and carotenoids, respectively [31]. SIPI increases when the vegetation is subject to a state of stress.

## 3. Results and Discussion

**Figure 3:**
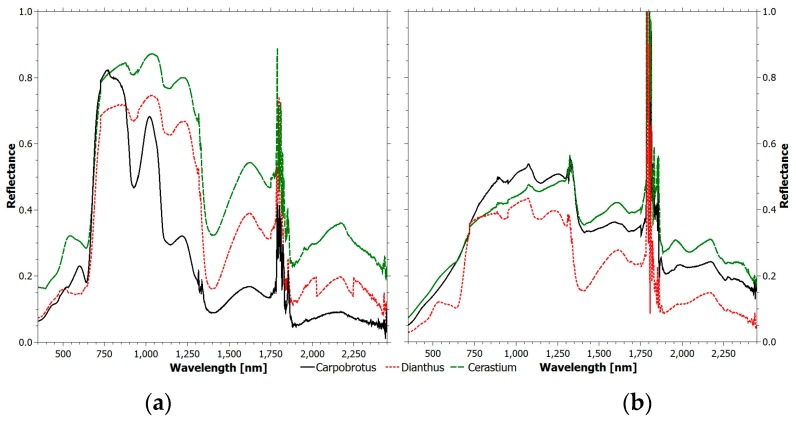
Comparison between the reflectance spectra of plants in (**a**) healthy and (**b**) unhealthy state.

Figure 3 shows a comparison between the spectra of plants in (a) healthy and (b) unhealthy state for the three species monitored in this study. Spectral reflectance values of the same plants were recorded at two different dates, i.e., 22 December 2014 and 11 May 2015. The spectral responses of the three species in the two situations are significantly different. The spectral signatures for the three species in health status reflect the typical behavior described in Section 2.3. It is worth noting that the green peak for Dianthus and Cerastium is at green wavelengths because healthy plants look green; instead, the Carpobrotus signature shows a peak at red wavelengths because when the plant is healthy it looks red. When the health status deteriorates, a significant increase of the red band reflectance occurs for both Carpobrotus and Cerastium. At the same time, the green peak disappears due to the decrease of light absorption caused by the diminishment of the chlorophyll content. The red edge slope decreases and its position moves toward lower wavelengths. The leaf maturation toward senescence sees a gradual decrease in the reflection in the NIR region due to cell deterioration [22].

**Figure 4:**
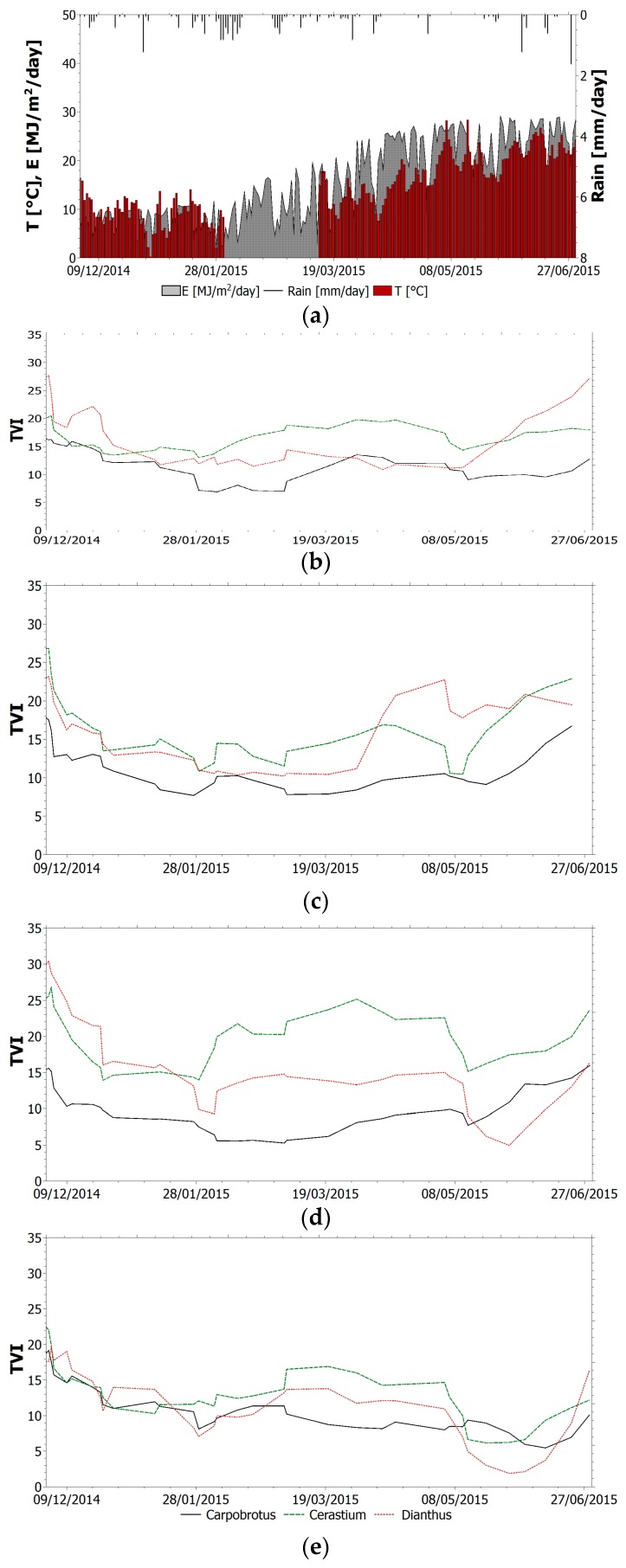
(**a**) Relevant meteorological parameters monitored during the acquisition period. TVI trend within the monitoring period for all species: (**b**) sector 1 upslope; (**c**) sector 1 downslope; (**d**) sector 2 upslope; (**e**) sector 2 downslope.

**Figure 5:**
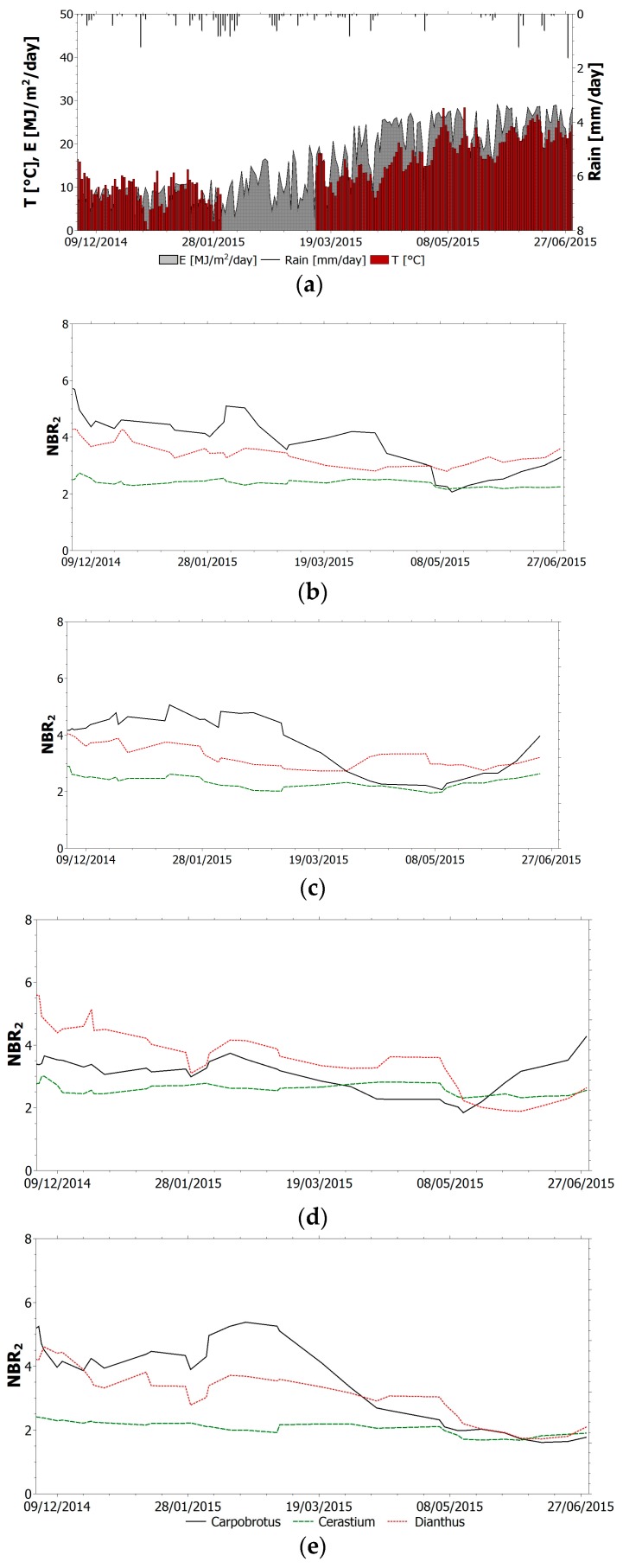
(**a**) Relevant meteorological parameters monitored during the acquisition period. NBR trend within the monitoring period for all species: (**b**) sector 1 upslope; (**c**) sector 1 downslope; (**d**) sector 2 upslope; (**e**) sector 2 downslope.

**Figure 6:**
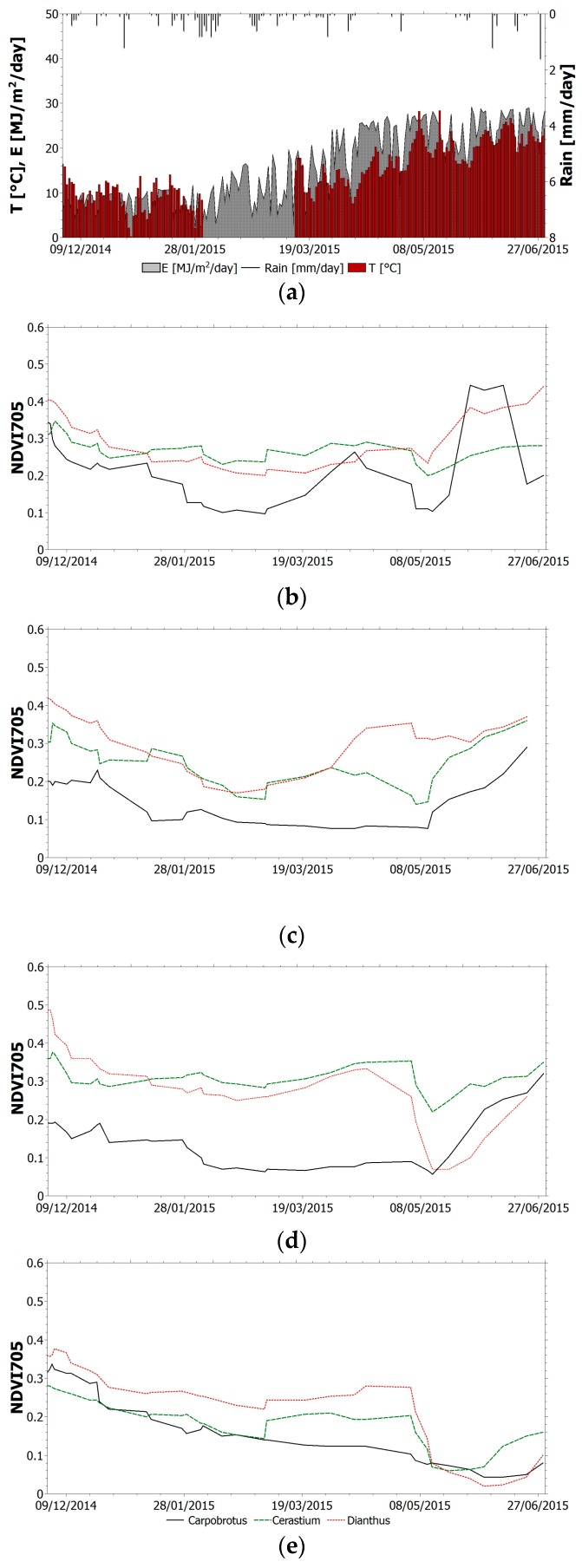
(**a**) Relevant meteorological parameters monitored during the acquisition period. NDVI_705_ trend within the monitoring period for all species: (**b**) sector 1 upslope; (**c**) sector 1 downslope; (**d**) sector 2 upslope; (**e**) sector 2 downslope.

**Figure 7:**
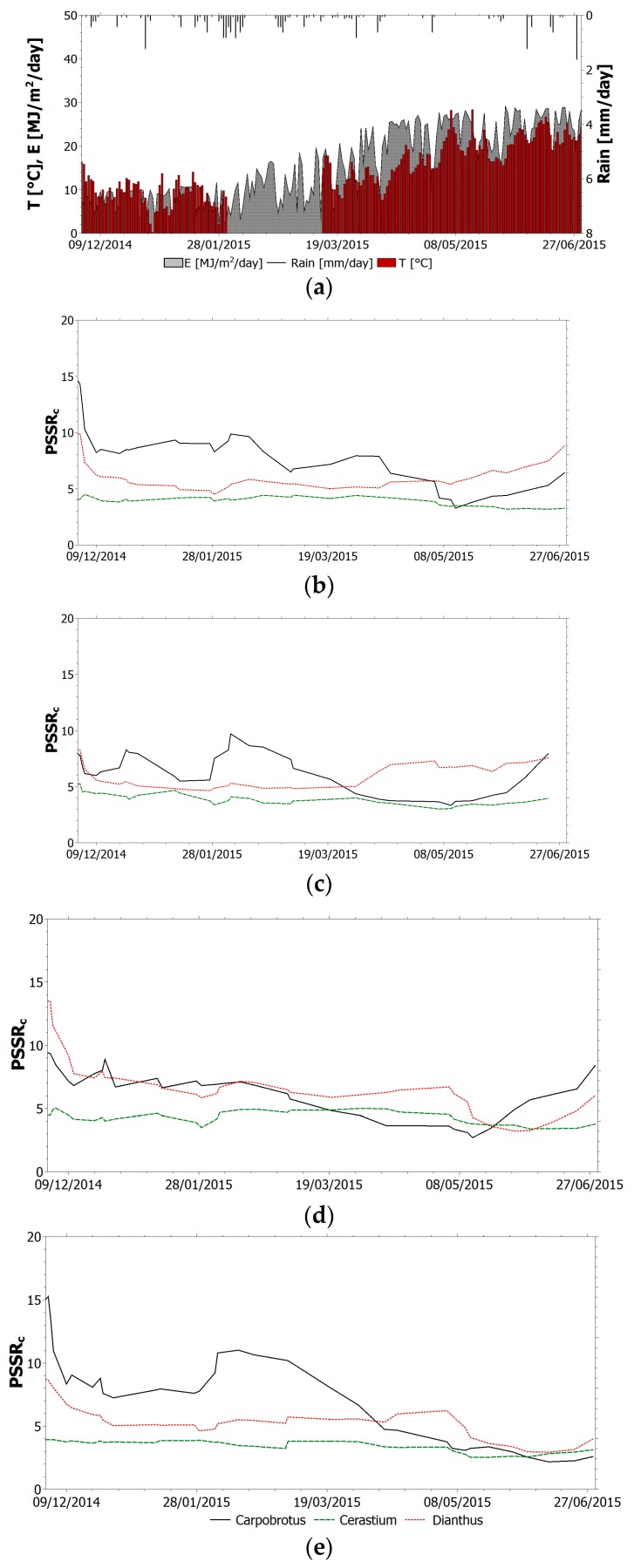
(**a**) Relevant meteorological parameters monitored during the acquisition period. PSSR trend within the monitoring period for all species: (**b**) sector 1 upslope; (**c**) sector 1 downslope; (**d**) sector 2 upslope; (**e**) sector 2 downslope.

**Figure 8:**
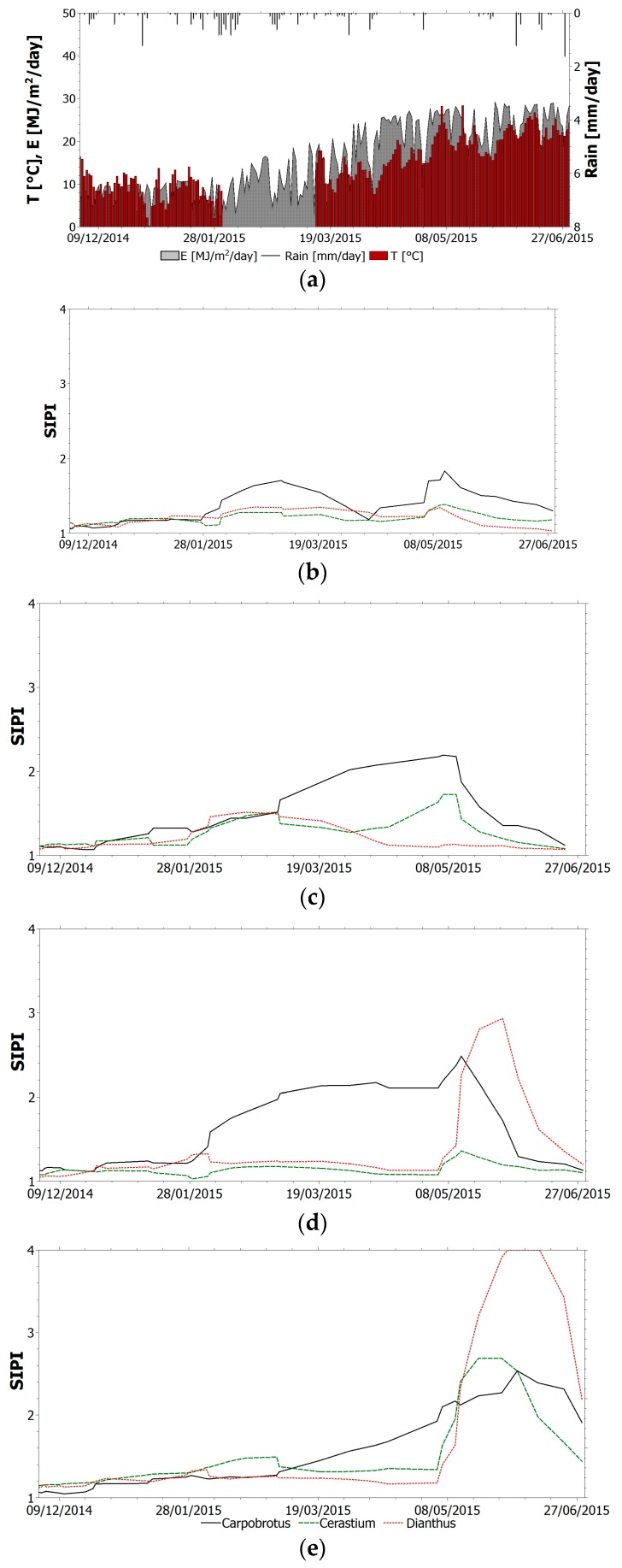
(**a**) Relevant meteorological parameters monitored during the acquisition period. SIPI trend within the monitoring period for all species: (**b**) sector 1 upslope; (**c**) sector 1 downslope; (**d**) sector 2 upslope; (**e**) sector 2 downslope.

The selected index (TVI, NBR, NDVI_705_, PSSR and SIPI) trend within the monitoring period for all species is shown in Figure 4, Figure 5, Figure 6, Figure 7 and Figure 8. It is worth noting that, in all figures, the spectral indices are presented together with the relevant meteorological parameters monitored during the acquisition period, such as solar radiation, air temperature and rainfall, collected through the meteorological station built on the roof. Furthermore, the *x*-axis represents the acquisition date, the *y*-axis the index value.

All trends reveal that there is an initial suffering period (November 2014–March 2015), shown by the decrease of indices related to chlorophyll content and by the increase of indices related to chlorophyll–carotenoid ratio. After this period, the vegetation health improves. The initial aggravation registered is connected to the winter season, where, as shown by monitored climate parameters, air temperature and solar radiation have low average values. Daily air temperature varies between 0 °C and 15 °C in December with an average value of 9 °C. Solar radiation during winter months is between 1 MJ/(m^2^day) and 16 MJ/(m^2^day). With the arrival of spring and better weather conditions, the general conditions in the green roof vegetation improve. Anomalous climate conditions were recorded in the first week of May, when air temperature rose to 28 °C and average daily solar radiation was 26.5 MJ/(m^2^day). High temperature and solar radiation values, with lack of precipitation, resulted in dryness and water shortage; this water stress is highlighted by the trend of all indices. To overcome this setback due to dryness and to prevent the plant dying, the sprinkler system on the roof was activated. The plants benefited from this and a clear change of the selected indices was recorded.

Generally speaking, all indices analyzed reflect the state of health of the plants, by exhibiting a certain degree of correlation with the eco-physiological state of the vegetation.

For the three species, TVI trend (Figure 4) shows an initial decrease that reflects a decrease in chlorophyll content due to wintertime. From February, the TVI starts to increase, testifying to an improvement of the plant health. In May, the index undergoes a reduction due to water stress, followed by a sudden increase related to the positive effects of the irrigation system activation on 8 May. By comparing the value of TVI for each species and for each sector, Cerastium appears as the healthier species on average compared to the other species (TVI values higher on average than for the other species) and it was affected by the local climatic conditions to a lesser extent than the Carpobrotus and Dianthus were. Conversely, Carpobrotus was the species that was most affected. These assessments reflect the real state of the plants observed during the entire acquisition period. No remarkable differences can be noticed for TVI trends in sector 1 and sector 2, and for plants located upslope or downslope.

By observing the NBR trend (Figure 5), it can be seen how, except for Cerastium, it reflects the above observations on the vegetation’s health during the monitoring period. In more detail, by analyzing individual trends, this index shows a good representation of health status for both Carpobrotus and Dianthus. After comparing NBR for these two species, the health state of Carpobrotus seems better than that of Dianthus. In fact, the index values for Carpobrotus are higher for sector 1 except for the period that goes from April to June 2015 and in sector 2 for plants located downslope. Conversely, NBR for Dianthus is higher than for Carpobrotus in sector 2 for plants located upslope. The index values do not correspond to the real state of the plants resulting from visual inspection during the entire acquisition period. This depends on the index definition and on the physiological difference between Carpobrotus and the other two species. In fact, NBR is a simple ratio index related to the reflectance in the green region of the electromagnetic spectrum. The green peak is remarkable as the plant looks green and healthy, but this does not go for Carpobrotus that shows red tones when the plant is in a healthy state (see Figure 3). The variation of the index values for Cerastium is significantly less marked; this means that the index is not suitable to provide information about the species health status.

The low reliability of NBR has been exceeded by NDVI_705_. The trend of this narrowband normalized index (Figure 6) shows a good correspondence with the real state of the vegetation previously described. Due to its mathematical definition, NDVI_705_ is more reliable than NBR. This is easily demonstrated by looking at the Carpobrotus trend: not only does the singular trend reflect the real state of the plant but also the comparison between the three species shows a good correspondence with the eco-physiological state of the green roof plants from visual inspection. In fact, during the monitoring period, Carpobrotus shows the most significant variations from its initial good conditions to a remarkable worsening of its health status up to the recovery period during summertime. No remarkable differences can be noticed for plants in sectors 1 and 2. This allows for the conclusion to be drawn that the thickness of the growing medium does not play a significant role in the plant’s health status. Conversely, indices for plants in sector 2 in the downslope location are always lower than for plants located upslope in the same sector. The same does not hold for sector 1. This may be attributed to the drainage system in sector 2 that is not efficient in removing water, which then affects the plant’s healthy status.

Among the chlorophyll–carotenoid ratio indices, the PSSR trend is difficult to interpret (Figure 7). Similarly to the other simple ratio index analyzed, PSSR for Cerastium shows a rather flat trend, which does not make it possible to get any useful information about the health state of the plant. In general, higher values of this index for Carpobrotus reflect an unhealthier state of the plant of this species with respect to the other ones. The index variations for this species indicate an initial increase in the carotenoid content in January 2015, a reduction with the arrival of spring and a further increase under hot climate conditions. The benefit related to the activation of the irrigation system is not entirely reflected by the index trend, with the conclusion that PSSR is not a suitable indicator of the eco-physiological vegetation state for the species investigated.

SIPI is a better stress plant indicator related to the chlorophyll–carotenoid ratio (Figure 8). By analyzing this index for the two sectors and for all the species investigated, it is evident that SIPI accurately reflects both the well-being and discomfort states of vegetation. In fact, its trend during the monitoring period testifies to the aggravation related to the cold season, the general improvement during springtime, the worsening caused by excessive heat (and the resulting water stress) and the sudden improvement after the activation of the irrigation system. Again, no remarkable differences can be noticed for sector 1 and 2. Conversely, the inefficacy of the drainage system in sector 2 is confirmed, as higher values of SIPI for plants located downslope are found.

## 4. Conclusions

Imaging spectroscopy of vegetation is a potentially useful tool to identify and prevent unhealthy conditions in plants. To the authors’ knowledge, its application to green roof monitoring has not yet been made. The system used has portability and high spectral resolution (1 nm) allowing great detail in the information extracted. Sampling is rapid, non-destructive, and applicable to scales from leaf to canopy.

Though the acquisition period appears limited in time, hyperspectral data acquisition and processing has triggered the definition of vegetation indices as useful plant health status indicators. A few vegetation indices were compared and their trends were thoroughly analyzed in relation to some meteorological parameters and the actual state of the plants resulting from visual inspection during the entire acquisition period. Broadband and narrowband indices related to chlorophyll content and to the chlorophyll–carotenoid ratio were compared as well as simple ratio, normalized difference and combinations of reflectance indices. The most representative health state indicators for sub-Mediterranean climate turned out to be NDVI_705_ and SIPI. In fact, thanks to its definition, the influenced structure of the plant was removed and the index revealed only the health state considering the leaf pigment content. The two indices turned out to be indicative of the plant stress due to water drought. The thickness of the growing medium does not play a significant role in the plant health status as shown by the comparison of the vegetation indices’ trends in sectors 1 and 2.

The preliminary results obtained with this investigation will be complemented in future investigations where the ground truth definition will be strengthened by: (i) collecting the leaf tissues for laboratory determination of the chlorophyll content and (ii) via chlorophyll meter.

## Figures and Tables

**Table 1:** Broadband and narrowband vegetation indices based on the content of chlorophyll and chlorophyll–carotenoid ratio. R denotes the reflectance, the subscript denotes the spectral bandwidth or the wavelength.

Broadband Index	Narrowband Indices	
*Chlorophyll Content*	*Chlorophyll Content*	*Chlorophyll–Carotenoid Ratio*
$\begin{array}{l} TVI=0.5(120(R_{NIR}-R_{GREEN})\\ \;-200(R_{RED}-R_{GREEN})) \end{array}$	$NBR=\frac{R_{750}}{R_{550}}$	$SIPI=\frac{R_{800}-R_{445}}{R_{800}-R_{680}}$
	$NDVI_{705}=\frac{R_{750}-R_{705}}{R_{750}+R_{705}}$	$PSSR=\frac{R_{800}}{R_{470}}$
